# Rhabdomyolysis After Total Abdominal Hysterectomy Requiring Urgent Hemodialysis Due to Hyperkalemia

**DOI:** 10.7759/cureus.14757

**Published:** 2021-04-29

**Authors:** Moeed Ahmed, Joseph Frederickson, Kanza Khan, Khalid Bashir

**Affiliations:** 1 Internal Medicine, Creighton University School of Medicine, Omaha, USA; 2 Internal Medicine, Services Institute of Medical Sciences, Services Hospital Lahore, Lahore, PAK; 3 Nephrology, Creighton University School of Medicine, Omaha, USA

**Keywords:** rhabdomyolysis, acute kidney injury, hyperkalemia

## Abstract

A 63-year-old woman with a past medical history of invasive ductal carcinoma of the breast, status post lumpectomy and chemoradiation, 15 cm left inguinal-femoral enlarged lymph node consistent with high-grade serous carcinoma of the ovary and 4.7 cm right adnexal mass underwent total abdominal hysterectomy, bilateral salpingo-oophorectomy, omentectomy, and lymph node dissection with cystoscopy and bilateral ureteral catheter placement. There was no intraoperative complication. After surgery, patient’s urine output decreased, and she developed acute kidney injury (AKI). Initially, it was thought that her reduced output might be due to surgery/anesthesia. She also developed arm and leg weakness raising suspicion for stroke. The neurological workup was unremarkable for any acute abnormality. Her creatinine kinase (CK) level was >20,000 u/l consistent with rhabdomyolysis. She was hydrated aggressively and required hemodialysis due to hyperkalemia. During the hospital course, her kidney function improved, and rhabdomyolysis resolved, and she did not require dialysis after discharge.

## Introduction

Rhabdomyolysis is a potentially lethal syndrome that involves necrosis of muscle cells and subsequent lysis and release of intracellular contents into plasma and urine. It can be associated with markedly elevated creatinine kinase (CK) level, myoglobinuria, hyperkalemia, hyperuricemia, hyperphosphatemia, cardiac arrhythmias, metabolic acidosis, compartment syndrome, and renal failure. Renal failure may occur as a potentially life-threatening subsequent complication in up to 46% of reported cases [1].

Rhabdomyolysis with subsequent myoglobinuria leading to acute tubular necrosis and acute kidney injury (AKI) was first described by Myer Betz in 1911. Though uncommon, rhabdomyolysis with AKI has been described as a post-surgical complication, most often after laparoscopic urological surgery and bariatric procedures due to prolonged positioning during surgery causing compression of muscles with resultant ischemia and reperfusion injury [2].

Early recognition of rhabdomyolysis in post-op patients can be difficult due to vague presentation. Timely recognition can prevent adverse consequences such as severe hyperkalemia and the need for dialysis.

## Case presentation

The patient is a 63-year-old woman with a past medical history of invasive ductal carcinoma of breast, status post lumpectomy and chemoradiation. The patient was admitted to the hospital for surgical management of a 15 cm left inguinal-femoral enlarged lymph node consistent with high-grade serous carcinoma of the ovary and 4.7 cm right adnexal mass. She underwent total abdominal hysterectomy, bilateral salpingo-oophorectomy, omentectomy, and lymph node dissection with cystoscopy and bilateral ureteral catheter placement. The procedure was done under general and spinal anesthesia. There was no intraoperative complication. Bilateral retrograde ureteral pyelograms were done and there was no evidence of ureteral injury or hydronephrosis. Ureteral catheters were removed. After the procedure, the patient was found to have low urine output. There was no urinary retention. Workup few hours after surgery revealed Hb 9.7 gm/dl, creatinine 1.5 mg/dl (baseline around 0.5 mg/dl), sodium 140 mmol/L, potassium 4.7 mmol/L, and bicarbonate 25 mmol/L. The patient was receiving lidocaine and ketamine infusion for pain management and ringer’s lactate (RL) for hydration. Ketamine drip was stopped as it was thought to be a possible cause of acute kidney injury and RL infusion rate was increased to 75 cc/hr. Further workup showed urine sodium 75 mmol/L, urine creatinine 42 mg/dl, and dipstick urinalysis was positive for blood, protein, red blood cells (RBCs), and white blood cells (WBCs). Renal ultrasound was unremarkable. On post-operative day 1, the patient developed hip pain, left arm weakness and numbness, and right leg weakness. Physical exam was remarkable for reduced strength in left arm and right leg. Vitals were: Temperature 36.5 °C, blood pressure 104/63 mmHg, heart rate 89 beats per min, respiratory rate 20 breaths per minute. Despite continuous intravenous hydration, the patient remained oliguric with urine output of less than 0.5 ml/kg/hr for several hours after surgery.

**Figure 1:**
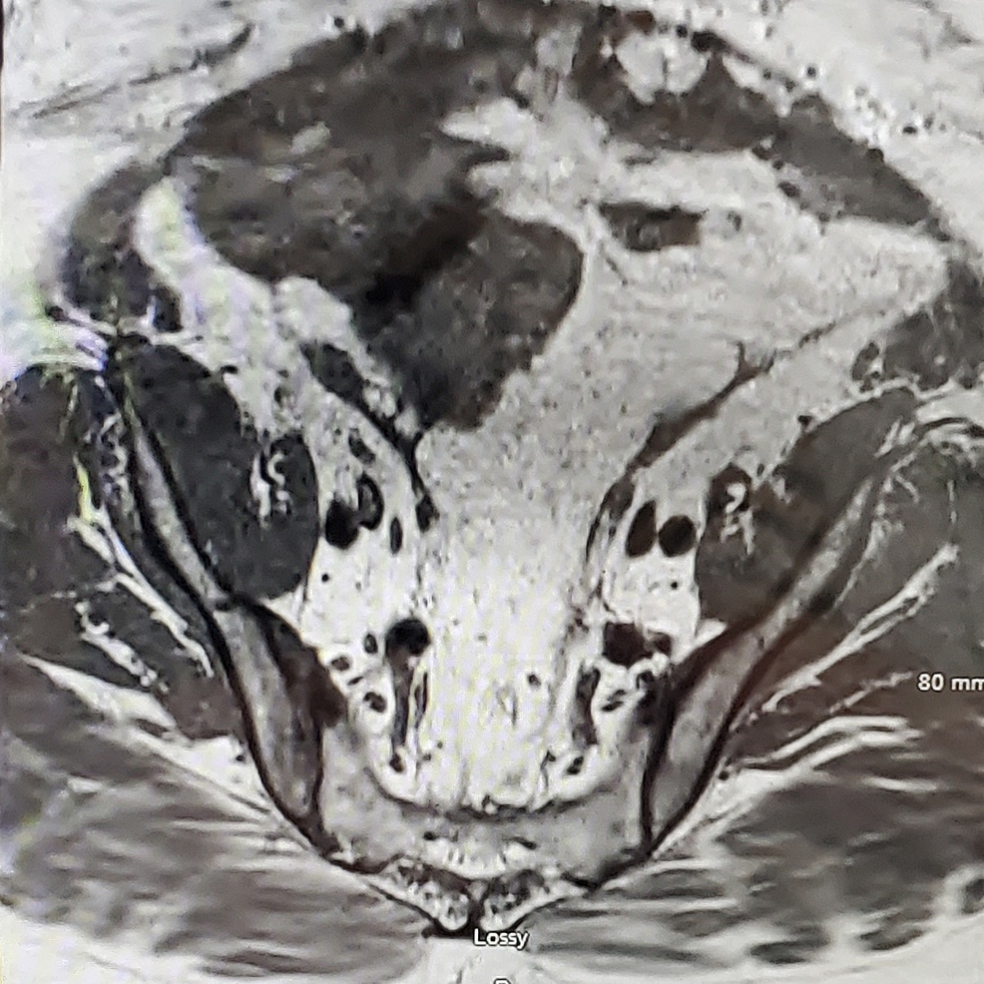
MRI pelvis

Given the weakness in the patient’s arm and leg, the neurology service was consulted, and a stroke workup was completed. MRI brain and MRI angiogram showed no evidence of acute stroke. MRI pelvis showed increased T2 signal/edema within the gluteus musculature and right adductor muscles (Figure 1).

**Figure 2:**
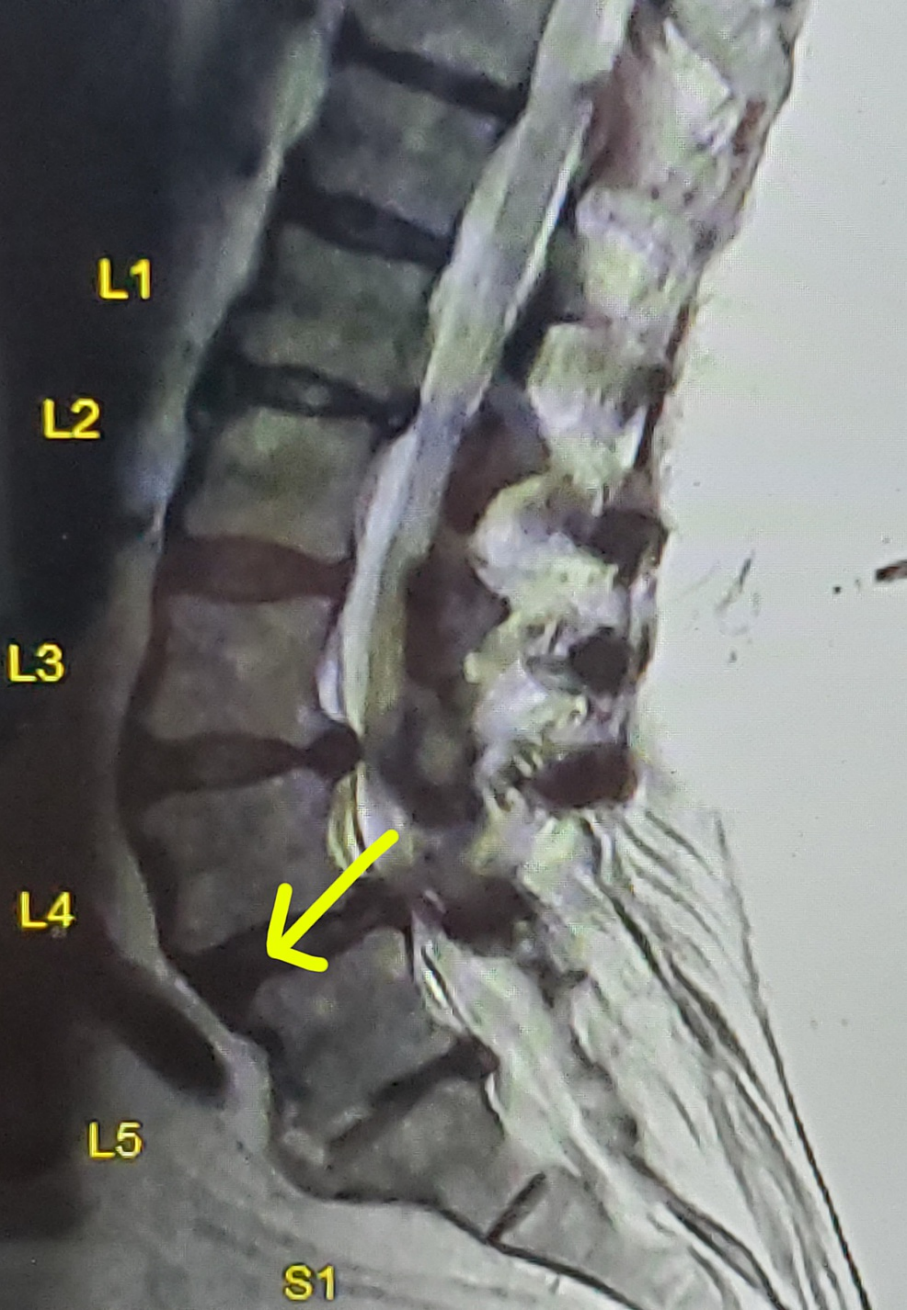
MRI lumbar spine

MRI lumbar spine showed severe spinal stenosis at L4-L5 without cord compression (Figure 2).

Creatinine kinase level was checked and was noted to be over 20,000 u/l. Phosphorus level was elevated at 6.5 mg/dl, and uric acid level was elevated at 8.8 mg/dl. The lab findings were consistent with rhabdomyolysis.

**Figure 3:**
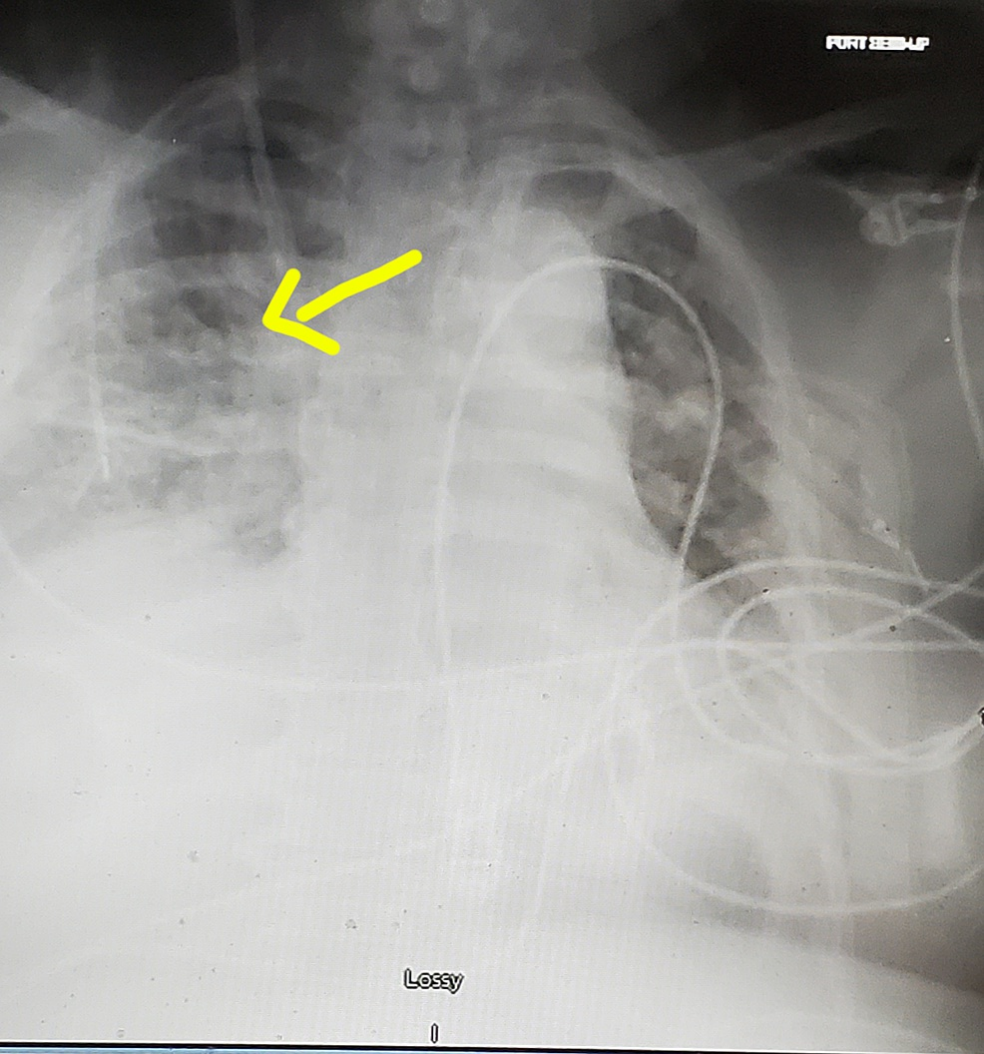
Chest X-ray

The patient was given aggressive intravenous fluids. Her creatinine continued to rise, and her urine output remained low despite aggressive hydration. Two days after surgery, potassium was elevated at 6.7 mg/dl, oxygen requirement had gone up, and chest X-ray showed pulmonary edema (Figure 3).

The patient underwent urgent hemodialysis with fluid removal. This was followed by another session of hemodialysis the next day.

The patient’s kidney function improved, and her rhabdomyolysis resolved in the hospital. She was discharged a week after admission with a CK level of 5186 u/l. Her creatinine peaked at 4.4 mg/dl on day 4 of hospitalization and came down to 1.14 mg/dl on day of discharge. A month later, she followed up with Nephrology in clinic. She reported urinating well and had a creatinine of 0.67 mg/dl indicating recovery from AKI.

## Discussion

Our case highlights the importance of early diagnosis of rhabdomyolysis. Our patient’s low urine output after surgery was initially thought to be due to anesthesia that led to the discontinuation of ketamine. The patient’s symptoms of arm and leg weakness raised suspicion for stroke and led to the MRI and MRI angiogram. It is reasonable to say that there was possibly a delay in diagnosing rhabdomyolysis that led to the hyperkalemia and need for dialysis.

Acute kidney injury as defined by the KDIGO clinical practice guidelines is a rise in serum creatinine of >0.3 mg/dl within 48 hours, a rise in serum creatinine >1.5 times baseline presumed to have occurred within the prior seven days, or urine volume <0.5 ml/kg/hr for six hours. A 2017 meta-analysis of post-operative AKI using data from the Veteran’s Health Administration found that 11.8% of patients hospitalized after major surgery developed an acute kidney injury and some studies have suggested that post-operative AKI may account for up to 40% of inpatient AKI cases [3]. The pathogenesis of AKI in these cases is multifactorial and often a result of diminished kidney perfusion as a result of pre-operative nothing by mouth orders, intraoperative blood loss, insensible fluid loss, and the effects of fluids extravasating into the tissues. This low effective arterial volume state activates the renin-angiotensin aldosterone system and decreases blood flow to the kidneys due to renal artery constriction [4]. Abdominal surgery in particular is associated with gut ischemia and resultant dysfunctional inflammatory cascades that are associated with tubular injury [4]. Anesthesia is thought to be a contributing factor due to its vasodilatory and myocardial effects. Surgery itself may also cause an increase in antidiuretic hormone (ADH) as a result of catabolic hormones. A retrospective cohort study at the University of Florida evaluated post-operative AKI in patients undergoing major gynecological surgery and found that, among this group of patients, major risk factors included: age, African-American ethnicity, a malignant rather than benign tumor, emergent vs elective surgery, weekend admission, metastatic disease, congestive heart failure, and chronic pulmonary disease. AKI among this group of patients was associated with longer hospital stays, admission to the ICU, and post-operative mechanical ventilation requirements [5].

Rhabdomyolysis appears to be an uncommon cause of post-operative AKI; however, prolonged operative times are associated with increased risk of rhabdomyolysis and subsequent AKI. Rhabdomyolysis occurs when striated muscle breaks down and releases its contents into the blood stream, in particular myoglobin. The etiology of myoglobin-induced kidney injury is not completely understood; however, it appears to be associated with direct tubular injury, obstruction, vasoconstriction, and ischemic tubular injury [1]. Patients who develop AKI associated with rhabdomyolysis develop electrolyte abnormalities, particularly hyperkalemia, that should be rapidly treated. Case series have demonstrated some benefit from the use of mannitol for diuresis that may be associated with increased excretion of heme pigment and may also act as a free radical scavenger. Bicarbonate may also be beneficial to alkalinize the urine for patients whose kidneys may be unable to do so. Studies suggest that aggressive volume resuscitation in these patients is generally sufficient. Mannitol and bicarbonate have not been demonstrated to provide benefit in large scale studies, so they are not generally recommended [3]. Dialysis itself is not effective at removing myoglobin from the bloodstream; however, some individuals may benefit from venous hemofiltration for removal of myoglobin in select cases [1].

A case of rhabdomyolysis after minimally invasive surgery for squamous cell carcinoma of the uterine cervix and adhesions due to deep infiltrating endometriosis has been reported [6].

The list of factors that can cause rhabdomyolysis is broad and included medications such as statin, alcohol, trauma, immobilization, infection, strenuous exercise, malignant hyperthermia, and neuroleptic malignant syndrome. Ketamine has been associated with rhabdomyolysis. In a case study of 20 patients with ketamine use, two developed rhabdomyolysis [7]. The home medication list of our patient did not include statin or any other high-risk medication for rhabdomyolysis. Our patient also had no significant history of alcohol use. Her temperature before and after surgery was within normal range. The long time spent in the procedure and recovery room was a potential cause of rhabdomyolysis in our patient. However, ketamine as a possible cause of rhabdomyolysis cannot be ruled out.

Some of the methods that can be used during surgery to prevent rhabdomyolysis include ensuring correct patient positioning every two hours, using intermittent pneumatic compression pumps for the lower extremities, and providing shoulder padding.

The potential causes of AKI in our case were anesthesia, hypovolemia, or urinary tract obstruction. Urinary tract obstruction was ruled out with pyelogram and ultrasound. Even after stopping ketamine drip and increasing intravenous hydration, urine output remained low indicating that anesthesia and hypovolemia were less likely culprits. The differential diagnosis of hip pain and arm and leg weakness included stroke/transient ischemic attack (TIA), spinal stenosis, and rhabdomyolysis. Although MRI revealed spinal stenosis, this could be chronic, and age related and there was no spinal cord compression and patient’s acute presentation did not seem to be consistent with spinal stenosis. The patient’s neurologic symptoms eventually subsided and due to their non-focal pattern and negative imaging workup, were attributed to local nerve injury. The elevated CK, uric acid, and phosphorus provided strong evidence to support a diagnosis of rhabdomyolysis. The treatment of rhabdomyolysis is aggressive intravenous hydration to increase oxygen delivery and dilute myoglobin and other renal tubular toxins. In contrast, the treatment for stroke/TIA is thrombolytic or antiplatelet and statin, and possible carotid endarterectomy.

## Conclusions

Our case emphasizes that rhabdomyolysis should be considered as a cause of AKI after surgery. Anesthesia and hypovolemia may cause low urine output after surgery but should not preclude a rhabdomyolysis workup. Proper positioning techniques should be used during surgery to prevent rhabdomyolysis.

## References

[1] Bosch X, Poch E, Grau JM (2009). Rhabdomyolysis and acute kidney injury. N Engl J Med.

[2] Pariser J, Pearce S, Patel S (2015). Rhabdomyolysis after major urologic surgery: epidemiology, risk factors, and outcomes. Urology.

[3] Grams M, Sang Y, Coresh J (2016). Acute kidney injury after major surgery: a retrospective analysis of Veterans Health Administration Data. Am J Kidney Dis.

[4] Gameiro J, Fonseca JA, Neves M, Jorge S, Lopes JA (2021). Acute kidney injury in major abdominal surgery: incidence, risk factors, pathogenesis and outcomes. Ann Intensive Care.

[5] Vaught AJ, Ozrazgat-Baslanti T, Javed A, Morgan L, Hobson CE, Bihorac A (2015). Acute kidney injury in major gynaecological surgery: an observational study. BJOG.

[6] Steinmacher S, Abele H, Brucker S, Taran F (2018). Case report on rhabdomyolysis after minimally invasive surgery for squamous cell carcinoma of the uterine cervix and adhesions due to deep infiltrating endometriosis. Case Rep Women's Health.

[7] Weiner AL, Vieira L, McKay CA, Bayer MJ (2000). Ketamine abusers presenting to the emergency department: a case series. J Emerg Med.

